# Concerns, Health Profiles, and Quality of Life of LGBTQIA+ Individuals in Maharashtra: A Mixed-Methods Study

**DOI:** 10.7759/cureus.106565

**Published:** 2026-04-07

**Authors:** Nidhi P Sastry, Jyotsna S Deshmukh, Taheseen Fatema Shaikh

**Affiliations:** 1 Community Medicine, Lokmanya Tilak Municipal Medical College and General Hospital, Mumbai, IND; 2 Community Medicine, Government Medical College, Nagpur, IND

**Keywords:** focus group discussion, lgbtqia+, mixed methods, quality of life, sexual orientation

## Abstract

Introduction: The abbreviation LGBTQIA+, short for lesbian, gay, bisexual, transgender, queer/questioning, intersex, and asexual, unites a wide range of gender and sexual identities that are frequently marginalized in society. Compared to their peers and coworkers from heterosexual groups, they have less access to public health facilities, which causes physical, mental, social, and financial issues. Hence, the objectives of the study are to explore the concerns and evaluate the health profile and quality of life of LGBTQIA+ individuals in Maharashtra.

Materials and methods: Our study employed an exploratory mixed-methods approach in a non-governmental organization (NGO) in Maharashtra. For the qualitative part, three focus group discussions (FGDs) were conducted, each with seven, seven, and six participants. In contrast, the quantitative part studied sociodemographic details, health profiles, concerns faced, and quality of life, using the World Health Organization Quality of Life - Brief Version (WHOQOL-BREF) scale among 200 self-identified individuals from the LGBTQIA+ community.

Results: Recurring concerns that emerged during FGDs were lack of family support, bullying, discrimination at schools and workplaces, and economic and legal problems. Psychological health was the most affected domain, with a mean score of 58.31 ± 6.48. The participants included 76 (38%) bisexual men, 23 (11.5%) bisexual women, 62 (31%) gay men, 11 (5.5%) lesbian women, and 24 (12%) individuals identifying as transgender. HIV/AIDS infection (73, 36.5%) was the most common morbidity. Transgender individuals were found to have lower levels of education, lower socioeconomic status, more discrimination faced at school and in the workplace (p < 0.001 each), and significantly lower quality of life (p = 0.002).

Conclusion: The present mixed-methods study highlighted significant disparities in health profile, quality of life, and lived experiences among the LGBTQIA+ community. The transgender community was seen to be significantly more impacted compared to others in terms of health, concerns faced, and quality of life.

## Introduction

LGBTQIA+ is an acronym that brings together many different gender and sexual identities that often face marginalization across society. The acronym stands for lesbian, gay, bisexual, transgender, queer, questioning, intersex, and asexual, and the "+" symbol represents the expanding spectrum of diverse gender identities and sexual orientations [1]. Another term used for them is "sexual minorities." Sexual minorities are a group whose sexual identity, orientation, or practices differ from the majority of the surrounding society [2]. Earlier, 'gay' was the broad term used to refer to sexual minorities, but the terminology has been expanded to lesbian, gay, bisexual, trans, queer, and intersex, among others [3]. They face several disparities when it comes to health and healthcare services. They have limited access to healthcare services compared to heterosexual populations. LGBTQIA+ people face tremendous difficulties growing up in a society where heterosexuality is often presented as the only acceptable orientation and homosexuality is stigmatized or socially disapproved [4].

Physical problems include high risk for developing sexually transmitted infections (STIs) and HIV/AIDS, addictions, undergoing hormonal therapy, and sex reassignment surgery (SRS) [2]. Mental problems include facing stigma, discrimination, and abuse in society; increasing the chances of suicide; lowered self-esteem; substance abuse; and poor quality of life. Gay and bisexual individuals are more likely to experience depression and anxiety than their heterosexual counterparts [4]. These emotions might include intense sadness, anxiety, loneliness, discomfort in social situations, and feeling overwhelmed. The discrimination and atrocities impact their self-confidence and self-esteem. Additionally, they are prone to school dropouts and homelessness, and also have difficulty in finding jobs and finding housing. Getting important documents like disability pensions, voter ID cards, ration cards, passports, and caste certificates is another social problem faced [2]. There also arises discrimination within the community. The lack of awareness and representation garners some pansexuals and asexuals the status of sexual minorities within the LGBTQIA+ community [5]. Emotional problems arise when LGBTQIA+ people are discriminated against at the workplace, which acts as a barrier to employment and financial stability.

The World Health Organization (WHO) defines quality of life as “an individual's perception of their position in life in the context of the culture and value systems in which they live and in relation to their goals, expectations, standards, and concerns” [6]. The discrimination, stigma, and lowered self-esteem due to their sexual orientation have been shown to reduce the quality of life among them. Very few studies have included the wide spectrum of this community or compared health aspects among different LGBTQIA+ subgroups. Hence, considering the above points and the need to understand and compare their problems, the qualitative part of the study aimed to explore the concerns of LGBTQIA+ individuals in Maharashtra, and the objectives of the quantitative part were to evaluate their health profile and assess the concerns and quality of life among LGBTQIA+ individuals in Maharashtra.

This work was previously presented as a paper presentation at the Maharashtra Chapter of the Indian Association of Preventive and Social Medicine (IAPSM) - Integrated Public Health Annual Conference 2026 in February 2026.

## Materials and methods

Study design, setting, and participants

Our study employed an exploratory mixed-methods approach, which was conducted in a non-governmental organization (NGO) in a city in Maharashtra for a duration of nine months from June 2024 to February 2025. The study participants included self-identified LGBTQIA+ individuals over 18 years of age who were willing to participate in the study and provide informed consent, while we excluded any participants who were unable to respond to the questionnaire due to severe illness or cognitive impairment.

Sample size estimation

For estimating the sample size of the quantitative part, a study conducted by Hebbar YRN et al. was taken as a reference [7]. The sample size (n) was calculated using the formula $n = \frac{z^2 p q}{e^2}$, where the prevalence of HIV infection among LGBT participants was 42.22%, and a margin of 7% error was considered. After substituting the values in the formula, the sample size derived was 191. The final sample size was rounded off to 200. For the qualitative part, 20 participants who volunteered to be a part of the study were selected.

Ethical considerations

Ethical approval was obtained from the Institutional Ethics Committee, Indira Gandhi Government Medical College, Nagpur, India (approval number: IGGMC/Pharmacology/IEC/2180-8/2024). Permission was taken from the president of the NGO. A list of LGBTQIA+ volunteers and visitors to the NGO for health services was obtained and selected for the study. 

Sampling and data collection

Qualitative Part

Three focus group discussions (FGDs) were carried out among 20 participants who volunteered to be a part of the study. Two FGDs included seven participants each, while the third FGD included six participants. FGDs were conducted till data saturation was reached. All interviews were conducted in Hindi for cultural sensitivity and participant engagement, then transcribed into English. Four groups of five were formed, and the concerns faced by them were discussed. The answers were noted and recorded through voice recording after verbal consent of all participants, and full confidentiality was maintained. 

Quantitative Part

For the quantitative part, further participants were enrolled through consecutive and snowball sampling till the sample size was achieved. The volunteers and patients visiting for health checkups were selected consecutively and were asked to refer further individuals from the community to complete the sample size. A predesigned, pretested pro forma was used for collecting information regarding sociodemographic details, health profile, concerns faced, and quality of life, which was assessed using the World Health Organization Quality of Life - Brief Version (WHOQOL-BREF) scale (Appendix A). The questions regarding the concerns of people were formed from the themes that emerged during FGDs. The WHOQOL-BREF is a 26-item instrument consisting of four domains: physical health (7 items), psychological health (6 items), social relationships (3 items), and environmental health (8 items); it also contains QOL and general health items. Each individual item of the WHOQOL-BREF is scored from 1 to 5 on a response scale, which is stipulated as a five-point ordinal scale. The scores are then transformed linearly to a 0-100 scale [8].

Statistical analysis

Qualitative Part

For the qualitative study, the data were analyzed using a thematic analysis method using transcripts from the FGDs. Audio-taped interviews were transcribed verbatim, and transcripts were repeatedly checked to avoid mistakes. Major ideas and themes that emerged after repeated reviews were chronicled and indexed as codes manually.

Quantitative Part

Data were filled in a Microsoft Excel spreadsheet (Microsoft Corp., Redmond, WA, USA) and analyzed by Jamovi software version 2.7.6 (The jamovi project, retrieved from https://www.jamovi.org). Quantitative data were expressed as mean and standard deviation, whereas qualitative data were expressed as numbers and percentages. The chi-square test and Fisher's exact test were applied to compare the categorical variables. The ANOVA test was applied after normality testing to compare the differences between the means of the groups. A p-value of < 0.05 was considered to be statistically significant.

## Results

Qualitative part

**Figure 1:**
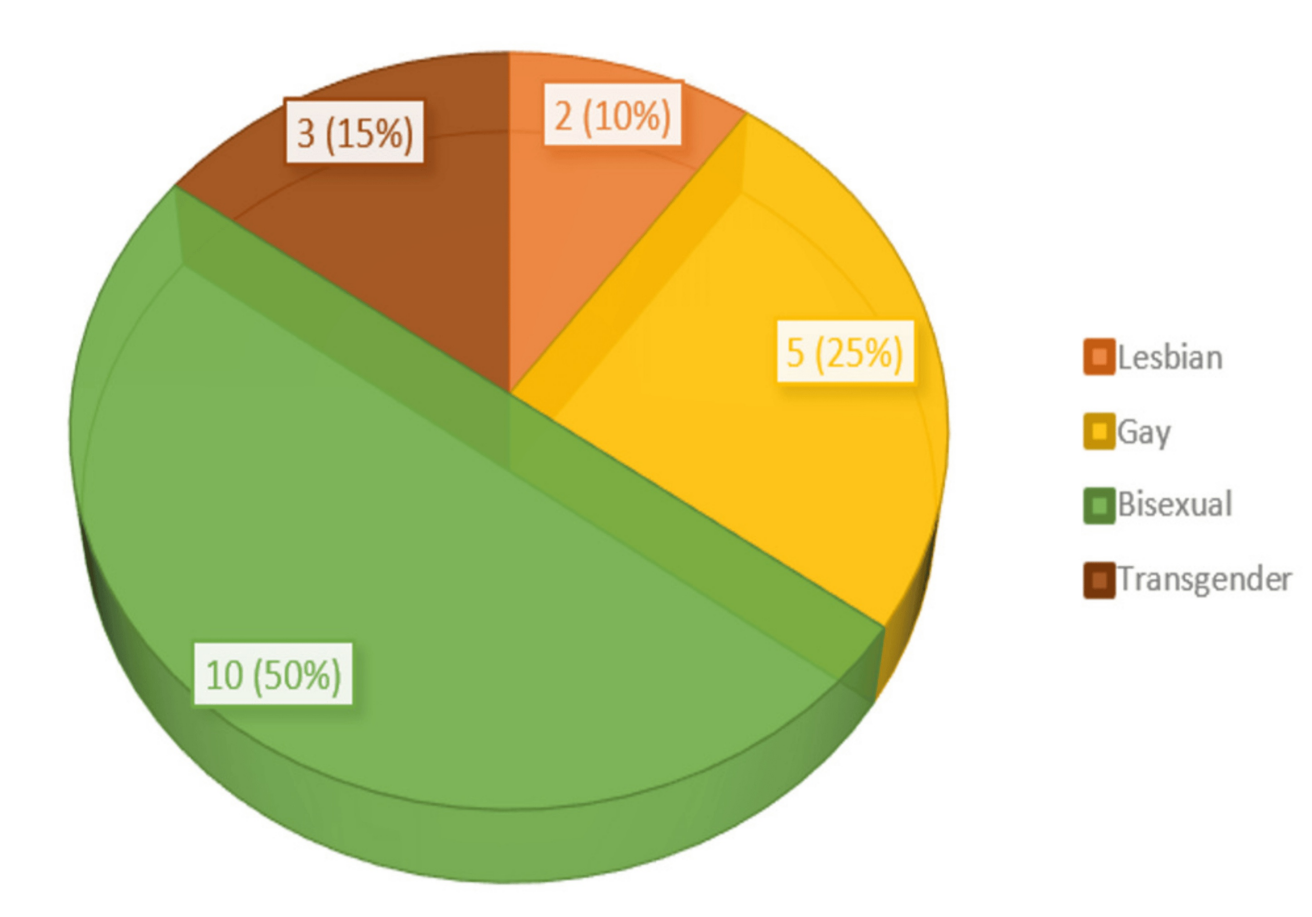
Sexual orientation of study participants (N = 20)

Figure 1 shows the sexual orientation of study participants included in the qualitative part of the study. The majority of participants in the FGD identified as bisexual (10, 50%), followed by those who identified as gay (5, 25%).

Table 1 shows various themes that emerged after the FGD regarding the concerns faced.

**Table 1:** Themes that emerged regarding concerns faced by the participants

Theme	Sub-themes
Problems faced in childhood	Acceptance of sexuality by oneself, acceptance of sexuality by family/friends, bullying/violence/lowered self-esteem faced in school
Lack of family support	Coming out to family, making family understand, support from family, abandonment by family
Workplace problems	Economic problems while finding a job, fewer job opportunities for some sexualities, and discrimination in the workplace
Issues faced at the hospital	Problems faced during form filling, during examination, no separate wards or washrooms, sex reaffirmation surgery cost, and follow-up
Legal problems faced	Legal issues, arrests from cruising sites, same sex marriage, adoption of a child while in same sex relationships

Problems Faced in Childhood

The first concern the participants faced was coming to terms with their sexuality themselves. Not having any reference point or anyone known led them to believe they were not normal or that what they thought was a sin. In the case of transgender participants, their references were the mass media, where transgender individuals are highly misrepresented, leading to them being scared and embarrassed about opening up. In school, they realized they were different when they stood out from the rest of the class in terms of preferences. Use of slang or foul language towards them was very common among boys. Words like “chhakka” and “baayla” were used to mock transgender individuals. The gay men in the study were bullied and shamed for not being interested in girls. They also faced violence on many occasions by fellow boys, which eventually lowered their self-esteem.

Lack of Family Support

The common concern among all participants was revealing their sexual orientation to the family and deciding whom to confront first. Most approached their mothers first, followed by siblings. The first step was making them understand that it is not an abnormal behavior or a health condition, but it is the gender identity of the person. Many family members refused to discuss further, while some tried to understand. Some participants in the FGD did manage to convince their family members, leading to them accepting their sexual orientation and gender identity, whereas some refused to accept them in society, breaking all ties with them. In the case of those identifying as bisexual, some approached their spouses, who later came along, but some have still hidden their sexual identity from their partners.

Workplace Problems

For participants who were gay, bisexual, or lesbian, finding work is comparatively easier. But they did face workplace discrimination if their sexual identity was revealed. Many were also terminated from jobs for the same reason, and low-paying jobs were the primary concern among them. For those not terminated, they were still uncomfortable in the setting due to constant judgment, indirect jokes, and taunts. All transgender participants in the study initially worked as beggars, and some even as sex workers, due to the preconceived notion of it being the only job avenue. But with time and development, they are being employed in better roles. One transgender participant in the study was also an activist fighting for the rights of transgender individuals. However, some hesitancy persists even now in most workplaces about hiring transgender people due to the stigma attached and also due to low educational backgrounds.

Issues Faced at the Hospital

The first point of contact is during the time of form filling. They feel excluded because forms in the hospital do not include any provision for sexual minorities. During visits to Integrated Counselling and Testing Centres (ICTC) centers in the past, the counsellors blamed and shamed the people from the community for having anal sex instead of first asking them about the same before forming opinions. Later, with the help of repeated counseling from the volunteers at NGOs, this kind of probing has stopped, and now open-ended questions regarding the use of condoms, etc., are asked. The next issue faced by transgender individuals was that they had no separate wards or no separate washrooms. The healthcare workers often tried to avoid attending to them. An incident was also narrated by them when a transgender person from their community was taken to a hospital, where the healthcare workers were unwilling to touch the patient to examine her, causing a delay in management and eventually worsening her condition.

Legal Problems Faced

The people from the LGBTQIA+ community face legal problems very frequently. Most of them are related to any violence faced by them from others. Another aspect is the cruising sites. Many community people go to cruising sites for networking and meeting more individuals like them, whereas some go to cruising sites as they have no other place where they feel accepted. A lot of times, they are arrested from these sites, but if our country had better provisions and destigmatization against them, they would be able to meet people in the open and socialize more formally. There are recurring visits to courts for passing any new provisions for their community. Many gay and lesbian couples are facing troubles due to same-sex marriages still not being legalized in India.

Some quotes from study participants are mentioned in Table 2. 

**Table 2:** Quotes from study participants LGBTQ: lesbian, gay, bisexual, transgender, queer/questioning

Quote	Quoted by
“First, I had to make myself aware of what changes were happening in my body. No one knew about my situation, so who would help me to educate myself about what was happening? So I started reading about transgender people, and then I educated my parents."	A participant who identified as transgender, 38 years old
“We are often harassed and physically abused at cruising sites by others. We are hit and assaulted because they think we will get scared easily. Not every person goes there to do something wrong. Many of us go to socialize or even form a community.”	A participant who identified as gay, 26 years old
“I had to agree to marry for my family’s sake. At that time, I was afraid to tell my parents how I felt about myself and my desires. So I married the woman they chose for me, and we have a child as well. I am still inclined to both men and women, but I am unable to share this with my wife, thinking of the society.”	A participant who identified as bisexual, 36 years old
"TV and other mass media show LGBTQ individuals in a wrong light or as a comedy quotient, because of which the public forms a wrong perception about our community. If they want to show our community, they should show our real lives and struggles, not as a subject for entertainment.”	A participant who identified as transgender, 45 years old

Quantitative part

Table 3 shows the sociodemographic details of the study participants. The sample included 76 (38%) participants who identified as bisexual men, 23 (11.5%) as bisexual women, 62 (31%) as gay men, 24 (12%) as transgender individuals, and 11 (5.5%) as lesbian women. Age distribution showed a significant difference (p < 0.001), with most participants identifying as lesbian women (11, 100%), gay men (48, 77.42%), and other gender/sexual minority groups (6, 100%) aged <40 years, whereas participants identifying as bisexual (59, 60.82%) and transgender (19, 79.17%) were predominantly >40 years.

**Table 3:** Sociodemographic characteristics of study participants Chi-square test, *Fisher's exact test

Sociodemographic characteristics	Lesbian (n = 11)	Gay (n = 62)	Bisexual (n = 97)	Transgender (n = 24)	Others (n = 6)	Total (n = 200)	P-value
Age group (Mean age: 33.05 ± 9.60 years; Range: 18 – 53 years)							
< 40 years	11 (100)	48 (77.42)	38 (39.18)	05 (20.83)	06 (100)	108	< 0.001*
> 40 years	0 (0.00)	14 (22.58)	59(60.82)	19 (79.17)	0 (0.00)	92	
Education							
Upto diploma	04 (36.36)	33 (53.23)	61 (62.89)	23 (95.83)	03 (50.00)	124	< 0.001
Graduation and above	07 (63.64)	29 (46.77)	36 (37.11)	01 (4.17)	03 (50.00)	76	
Occupation							
Legislators, senior officials, and managers	05 (45.45)	01 (1.61)	04 (4.12)	2 (8.33)	0 (0.00)	12	< 0.001*
Technicians and associate professionals	2 (18.18)	16 (25.81)	20 (20.62)	7 (29.17)	3 (50.00)	48	
Clerks	0 (0.00)	3 (4.84)	14 (14.43)	0 (0.00)	0 (0.00)	17	
Skilled workers, shop workers	0 (0.00)	19 (30.65)	17 (17.53)	0 (0.00)	0 (0.00)	36	
Craft and related trade workers	0 (0.00)	1 (1.61)	9 (9.28)	0 (0.00)	0 (0.00)	10	
Plant and machine operators and assemblers	0 (0.00)	1 (1.61)	5 (5.15)	4 (16.67)	0 (0.00)	10	
Elementary occupation	2 (18.18)	21 (33.87)	24 (24.74)	11 (45.83)	0 (0.00)	58	
Homemakers	0 (0.00)	0 (0.00)	4 (4.12)	0 (0.00)	0 (0.00)	4	
Students	2 (18.18)	0 (0.00)	0 (0.00)	0 (0.00)	3 (50.00)	5	
Socioeconomic status (Modified B.G Prasad Scale)							
Class I	7 (63.64)	3 (4.84)	10 (10.31)	2 (8.33)	3( 50.00)	25	< 0.001*
Class II	1 (9.09)	11 (17.74)	13 (13.40)	3 (12.50)	1 (16.67)	29	
Class III	1 (9.09)	24 (38.71)	42 (43.40)	5 (20.83)	2 (33.33)	74	
Class IV	1 (9.09)	19 (30.65)	29 (29.90)	9 (37.50)	0(0.00)	58	
Class V	1 (9.09)	5 (8.06)	03 (3.09)	5 (20.83)	0(0.00)	14	

Educational status also differed significantly (p < 0.001), with most transgender individuals (23, 95.83%) and bisexual participants (61, 62.89%) educated up to diploma level, while a higher proportion of lesbian participants (7, 63.64%) had graduation and above.

Occupational distribution showed significant variation (p < 0.001); lesbian individuals were more commonly legislators/senior officials (5, 45.45%), whereas gay men (21, 33.87%), bisexual individuals (24, 24.74%), and transgender individuals (11, 45.83%) were mainly in elementary occupations.

Socioeconomic status differed significantly (p < 0.001), with lesbian individuals in the upper classes, while the lower classes constituted more transgender individuals, gay, and bisexual individuals.

**Figure 2:**
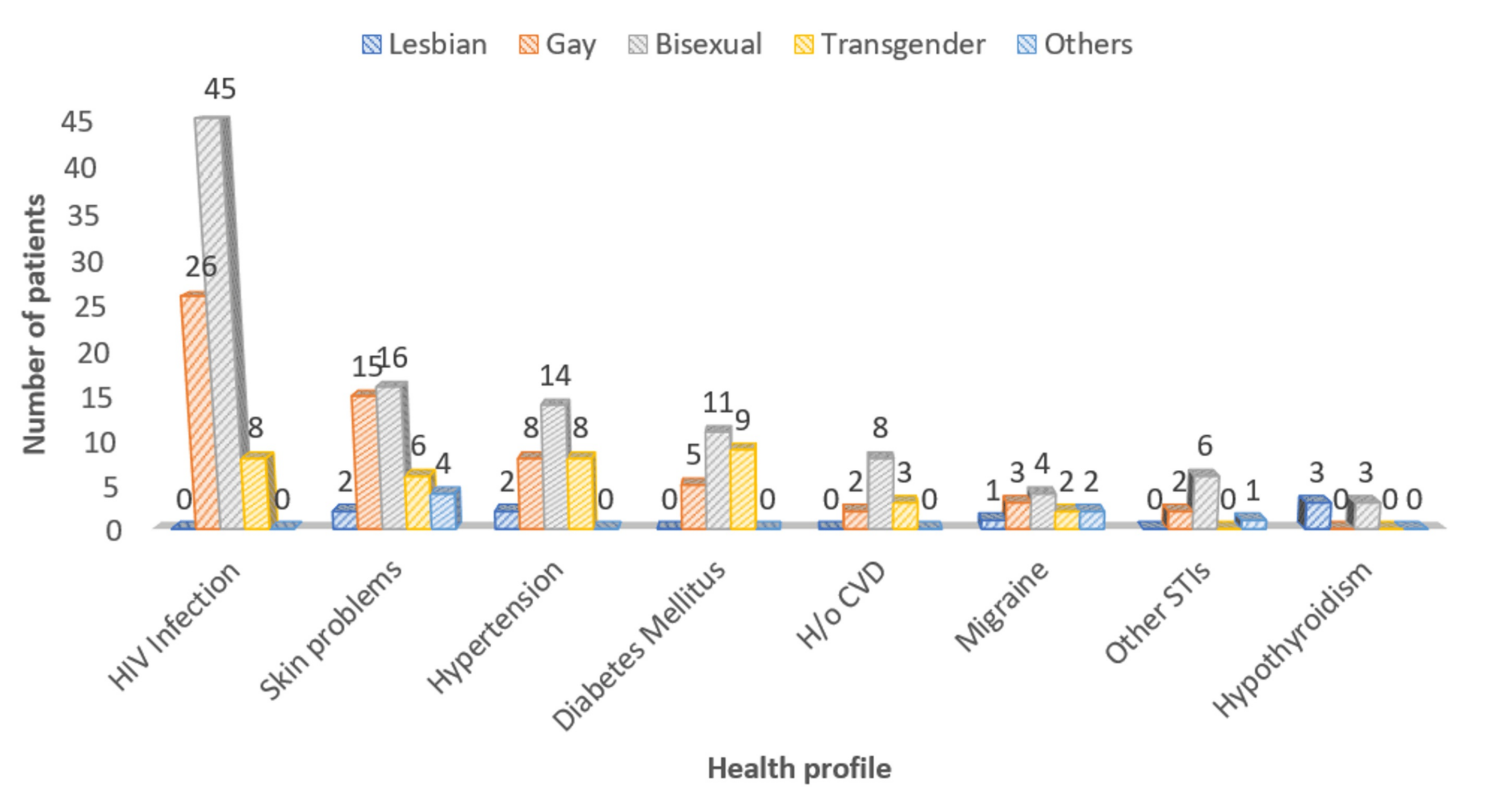
Health profile of the study participants STIs: sexually transmitted infections; H/o CVD: history of cardiovascular disease

As observed in Figure 2, among the 200 participants, HIV infection was the most common morbidity, reported in 79 (39.5%) participants, including 45 (22.5%) participants who identified as bisexual, followed by 26 (13%) who identified as gay and eight (4%) transgender participants. This was followed by skin problems, hypertension, and diabetes mellitus, reported among 43 (21.5%), 32 (16%), and 25 (12.5%) participants, respectively.

Table 4 shows the health behaviors of the study participants. A history of tobacco chewing was more common among participants who identified as gay (19, 30.65%) and those in other categories (2, 33.33%), although the difference was not statistically significant (p = 0.118). Alcohol use was most common among participants who identified as gay (27, 43.55%) and bisexual (30, 30.93%), followed by transgender participants (5, 20.83%), lesbian participants (3, 27.27%), and those in other categories (1, 16.67%) (p = 0.239). Condom use differed significantly across groups (p < 0.001), being highest among transgender participants (24, 100%) and participants who identified as gay (56, 90.32%), compared to those who identified as bisexual (43, 55.13%) and those in other categories (3, 50%). Among transgender participants, 13 out of 24 (54.17%) had undergone gender-affirming surgery and were receiving hormone replacement therapy.

**Table 4:** Distribution of study participants according to their health behavior Chi-square test, *Fisher's exact test

Health behaviour	Lesbian (n = 11)	Gay (n = 62)	Bisexual (n = 97)	Transgender (n = 24)	Others (n = 6)	Total (n = 200)	P-value
History of tobacco chewing	2 (18.18)	19 (30.65)	22 (22.68)	1 (4.17)	2 (33.33)	46	0.118
History of smoking	2(18.18)	20 (32.26)	22 (22.68)	1 (4.17)	2(33.33)	47	0.086
History of alcohol use	3 (27.27)	27 (43.55)	30 (30.93)	5 (20.83)	1 (16.67)	66	0.239*
Condom use	--	56 (90.32)	43 (44.39)	24 (100)	3 (50)	126	< 0.001

Table 5 shows the concerns faced by study participants. The variables were selected based on findings from focus group discussions. Compared to other groups, transgender participants reported significantly higher levels of social and structural challenges (p < 0.001). All transgender participants reported difficulty in disclosing their sexuality (24, 100%), which was higher than participants who identified as lesbian (3, 27.27%), gay (44, 70.97%), bisexual (50, 51.55%), and other categories (4, 66.67%). Only transgender participants reported a compulsion to engage in sex work (20, 83.33%) and begging (21, 87.5%), while none of the other groups reported these issues. Overall, the transgender group, followed by participants who identified as gay, experienced significantly more concerns than the remaining groups.

**Table 5:** Distribution of study participants according to the concerns faced Chi-square test, *Fisher's exact test

Concerns faced	Lesbian (n = 11)	Gay (n = 62)	Bisexual (n = 97)	Transgender (n = 24)	Others (n = 6)	Total (n = 200)	P-value
Coming out about their sexuality	3 (27.27)	44 (70.97)	50 (51.55)	24 (100)	4 (66.67)	125	< 0.001*
Compulsion to engage in sex work	0(0.00)	0(0.00)	0(0.00)	20 (83.33)	0 (0.00)	20	< 0.001*
Compulsion to work as a beggar	0(0.00)	0(0.00)	0(0.00)	21(87.5)	0 (0.00)	21	< 0.001*
Bullying/violence faced in school/workplace	1 (9.09)	58 (93.55)	32 (32.99)	24 (100)	4 (66.67)	119	< 0.001*
Discrimination faced in school/workplace	0 (0.00)	40 (64.53)	20 (20.62)	24 (100)	4 (66.67)	88	< 0.001*
Problems faced while visiting a hospital	0(0.00)	32 (51.61)	6 (6.19)	22 (91.67)	3 (50.00)	63	< 0.001*
Economic problems faced	7 (63.64)	40 (64.52)	58 (59.79)	19 (79.17)	1 (16.67)	125	0.075*
Legal issues faced	3 (27.27)	21 (33.87)	5 (5.15)	19 (79.17)	3 (50.00)	51	< 0.001*

Table 6 depicts the quality of life of study participants across four domains using the WHOQOL-BREF scale. Raw scores were measured, and then total scores were taken. Participants had overall better scores in the physical health domain, followed by the social relationships domain. The lowest score was for psychological health (58.31 ± 6.48).

**Table 6:** Domain-wise quality of life scores of the study participants

Domains	Score (Mean ± SD)
Physical health	69.50 ± 7.63
Psychological health	58.31 ± 6.48
Social relationships	67.43 ± 8.30
Environment	60.34 ± 7.18
Total score	63.89 ± 3.79

Table 7 shows the association between sexual orientation and the quality of life of the study participants. The scores have been expressed as mean and standard deviation; it can be observed that transgender individuals showed lower total scores compared to the rest. When the overall association was analyzed, a significant association was found between the mean quality of life score and sexual orientation (p = 0.002). 

**Table 7:** Association between sexual orientation and quality of life ANOVA test

Sexual orientation	Total Score (Mean ± SD)
Lesbian	66.05 ± 5.68
Gay	63.06 ± 3.56
Bisexual	64.14 ± 3.26
Transgender	61.32 ± 4.10
Others	64.58 ± 5.29
F stat	4.48
p-value	0.002

## Discussion

Qualitative part

In our study, several families have a preconceived notion that being gay or transgender is an abnormal behavior or just some peer pressure. Similarly, in a study by Bowling et al., participants mentioned their identity not being taken seriously by their family or being turned into a joke, or thought it was a “phase” [9]. This is mainly due to a lack of awareness or a genuine reference point for families to look up to or get themselves educated on. Lack of work opportunities led the transgender people to work as sex workers or as beggars, and similar observations were made by Chettiar in their study, where most of them were engaged in these two professions and often faced harassment by police for the same [10].

Participants in the current study faced problems in the hospital in terms of discrimination by healthcare workers, and the same was also seen in studies by Sharma et al. and Hunt et al., where the healthcare workers had misconceptions regarding the people from the community, which often led to discrimination while attending to them in clinics [11, 12].

The Supreme Court of India also extended its hand and provided relief to the LGBTQ community in its two pathbreaking judgments. The first one is that it recognized "transgender" as the third gender besides "male" and "female" and passed a slew of guidelines to all citizens of the country to adopt inclusionary practices at public and private places with the LGBTQ community [11]. However, even today, there is stigma attached to hiring them in dignified professions, and the facilities for a third gender, like separate hospital wards and separate toilets, are still not available.

Quantitative part

The current study employed a mixed-methods approach to assess the health profile and quality of life of participants and to explore the concerns faced by them. Most of the participants in this study were males (142, 71%). This skewed gender distribution could be because females are still not willing to open up about their sexual orientation publicly, and hence, they were also fewer in number at the NGO. Major participation in our study was by bisexual individuals (97, 48.5%), followed by gay individuals, then transgender participants, and only 11 (5.5%) lesbian participants. Contrary to our findings, Longna et al. had a majority of the participants by gay individuals, followed by lesbian individuals [13]. The majority of our study participants had studied up to the intermediate level or diploma (79, 38.5%), whereas in a study by Sucharitha et al., the majority had completed high school [14].

The prevalence of tobacco users (smoking and smokeless) was lower in our study when compared to Sucharitha et al. [14]. The lower number of people could be due to social desirability bias or could also be attributed to the fact that they are counselled by the NGO staff during every visit. The prevalence of HIV/AIDS infection in the current study was 36.5%, which was less than the study by Hebbar and Singh [7]. Thirteen out of 24 transgender individuals had undergone sex reaffirmation surgery along with hormone replacement therapy. No gay, lesbian, or bisexual person in our study had undergone the same, similar to findings by Vashisht et al. [15]. Reasons for not being able to undergo the surgery were financial constraints.

Our findings indicated that psychological well-being was the most adversely affected aspect among the participants. Mental health inequalities among the LGBT population were also noted by Williams [16]. The scores were particularly low among transgender individuals, followed by gay participants. This suggests a greater psychological burden in these groups, possibly due to stigma, discrimination, and social marginalization faced by sexual minorities. These findings are consistent with the study by Ghosh and Paliwal and other studies, which also reported poorer psychological quality of life among transgender individuals [17,18]. Societal rejection, lack of family support, and barriers in accessing healthcare and employment further contribute to poorer psychological health among transgender populations [19, 20].

The strengths of the study are that it includes the entire spectrum of the LGBTQIA+ population and tries to understand their physical, psychological, economic, and social problems collectively. We have studied many new aspects that have not been studied in the past, across the entire spectrum. Being a mixed-methods study, the current research explored concerns faced by them in depth. The limitations are that there is a possibility of social desirability bias while revealing answers related to their health behavior. An equal or proportionate representation across various sexual orientations would have given us a better picture, but we had fewer people from the lesbian and transgender community in the study as compared to gay and bisexual individuals, which could affect some variables of the study. The representation of lesbians was lower in the qualitative study as well, as not many wanted to come forward regarding their sexual orientation. Furthermore, since this was a single NGO-based study in one state, more studies across other study settings will be required for the findings to be generalizable. 

## Conclusions

The present mixed-methods study highlights significant disparities in health profile, quality of life, and lived experiences among the LGBTQIA+ community. Qualitative findings revealed recurrent themes of discrimination, bullying, lack of family support, workplace exclusion, healthcare-related stigma, and legal harassment, all of which negatively influence well-being. HIV infection emerged as the most common morbidity, emphasizing continued vulnerability to sexual health risks. The transgender community was seen to be significantly more impacted compared to others in terms of health, concerns faced, and quality of life.

More studies among the LGBTQIA+ community need to be conducted across our country with more participation of females and transgender persons. The mass media has an important role to play in changing the perceptions of people about the community and a more culturally sensitive representation of it. Sensitization of healthcare professionals, human resources, policemen, and teaching faculty at schools and colleges against discrimination would enable awareness creation at lower levels that could help put an end to the violence and bullying faced. Equal and stigma-free work opportunities should be provided, especially to transgender and gay individuals. Financial support for sex reassignment treatments should be made available.

## Appendices

Appendix A

**Table 8:** Study proforma

Sr. no	Question	Answer
1.	Age (years)	
2.	Age at which you came in terms with your sexuality	
3.	Gender	Male / Female / Transgender / Non binary / Gender fluid
4.	Sexual orientation	Lesbian / Gay / Bisexual / Transgender / Intersex / Queer / Questioning / Asexual / Auto sexual / Pansexual / Agender / Greysexual / Demisexual / Sapiosexual / Others
5.	Education	Illiterate/Primary/Secondary/Intermediate/ Diploma/ Graduate/Post graduate
6.	Occupation	Legislators, senior officials and managers Technicians and associate professionals Clerks Skilled workers, shop workers Craft and related trade workers Plant and machine operators and assemblers Elementary occupation Homemakers Students
7.	Socioeconomic class	I/II/III/IV/V
8.	Habit of tobacco/alcohol/smoking	Yes/No If yes, which _____________
9.	History of condom use	Yes / No / Not applicable
10.	Any morbidities	
11.	Have you undergone any sex reaffirmation surgery?	Yes / No / Not applicable
12.	Have you taken hormonal replacement therapy?	Yes / No / Not applicable
13.	Have you come out to your family?	Yes / No
14.	If yes, have they accepted your sexuality/gender identity ?	Yes / No / Neutral
15.	Was there any compulsion to go for sex work?	Yes/No
16.	Was there any compulsion to work as a beggar?	Yes / No
17.	Have you faced bullying/violence in school/workplace?	Yes / No
18.	Have you faced discrimination in school/workplace?	Yes / No
19.	Have you faced problems while visiting a hospital?	Yes / No
20.	Have you faced any economic problems?	Yes / No
21.	Have you faced legal problems?	Yes / No
